# “Systematic Review of Brief Cognitive Screening Tools for Identifying Dementia and Mild Cognitive Impairment in Illiterate and Low‐Educated Populations”

**DOI:** 10.1002/alz.092576

**Published:** 2025-01-03

**Authors:** Jonathan Adrian Zegarra‐Valdivia, Brenda Nadia Chino‐Vilca, Kuripacha Alcamari Tituana, Lina Maria Zapata‐Restrepo, Maria M. Unaucho Pilalumbo, Milton Gerardo de Jesus Lopez Norori, Carmen Paredes Manrique, Nilton Custodio

**Affiliations:** ^1^ Universidad Señor de Sipán, Chiclayo Peru; ^2^ Laboratory of Cognitive and Computational Neuroscience, Center for Biomedical Technology, Universidad Complutense and Universidad Politécnica de Madrid, Madrid Peru; ^3^ Achucarro Basque Center for Neuroscience, Bilbao Spain; ^4^ Global Brain Health Institute, San Francisco, CA USA; ^5^ Facultad de Ciencias de la Salud, Universidad Icesi, Cali, Colombia, Cali Colombia; ^6^ Global Brain Health Institute ‐ UCSF, San Francisco, CA USA; ^7^ Fundacion Valle del Lili, Cali Colombia; ^8^ Global Brain Health institute, San Francisco, CA USA; ^9^ Fundación Alzheimer de Nicaragua., Managua Nicaragua; ^10^ UCSF/GBHI, San Francisco, CA USA; ^11^ Universidad Tecnológica del Perú, Arequipa Peru; ^12^ Escuela Profesional de Medicina Humana, Universidad Privada San Juan Bautista, Lima, LIMA Peru; ^13^ Instituto Peruano de Neurociencias, Lima, Lima Peru

## Abstract

**Background:**

The increasing prevalence of dementia, particularly in low‐income regions, necessitates the development of effective cognitive screening methods. However, the suitability of many existing tools for illiterate and low‐educated populations is limited, resulting in significant diagnostic disparities. This review aims to evaluate global cognitive assessment instruments for the identification of Mild Cognitive Impairment (MCI) and dementia in older adults with limited education.

**Method:**

Following the guidelines outlined by PRISMA (Preferred Reporting Items for Systematic Reviews and Meta‐Analyses), a systematic review was conducted. This review encompassed searches across three databases for studies published between 2000 and 2023, with a focus on individuals aged 45 and above. Out of 1511 studies screened, 26 studies met the predefined inclusion and exclusion criteria.

**Result:**

Numerous studies have endeavored to adapt existing cognitive assessment tools to local languages to suit low‐literacy populations. Within this group, twelve distinct tests were identified. Notably, while the Mini‐Mental State Examination (MMSE) and the Montreal Cognitive Assessment (MoCA) are frequently used, they exhibit a notable bias towards education levels. The Rowland Universal Dementia Assessment Scale (RUDAS) exhibited strong performance, particularly among individuals with limited education, displaying high sensitivity and specificity.

**Conclusion:**

In summary, adjusting cutoff scores can be a valuable strategy for assessing individuals with limited education, but the development of customized cognitive assessment tools is imperative. The creation of region‐specific, sensitive cognitive evaluations, encompassing functional assessments, is pivotal to achieving a comprehensive diagnostic evaluation.

